# Mitochondrial Aconitase and Its Contribution to the Pathogenesis of Neurodegenerative Diseases

**DOI:** 10.3390/ijms25189950

**Published:** 2024-09-15

**Authors:** Volodymyr Padalko, Filip Posnik, Malgorzata Adamczyk

**Affiliations:** 1 Laboratory of Systems and Synthetic Biology, Chair of Drug and Cosmetics Biotechnology, Faculty of Chemistry, Warsaw University of Technology, Noakowskiego 3, 00-664 Warsaw, Poland; 2 School of Medicine, V. N. Karazin Kharkiv National University, 61022 Kharkiv, Ukraine

**Keywords:** mitochondrial aconitase, neurodegenerative disorders, oxidative stress

## Abstract

This survey reviews modern ideas on the structure and functions of mitochondrial and cytosolic aconitase isoenzymes in eukaryotes. Cumulative experimental evidence about mitochondrial aconitases (Aco2) as one of the main targets of reactive oxygen and nitrogen species is generalized. The important role of Aco2 in maintenance of homeostasis of the intracellular iron pool and maintenance of the mitochondrial DNA is discussed. The role of Aco2 in the pathogenesis of some neurodegenerative diseases is highlighted. Inactivation or dysfunction of Aco2 as well as mutations found in the ACO2 gene appear to be significant factors in the development and promotion of various types of neurodegenerative diseases. A restoration of efficient mitochondrial functioning as a source of energy for the cell by targeting Aco2 seems to be one of the promising therapeutic directions to minimize progressive neurodegenerative disorders.

## 1. Introduction

In modern society, neurodegenerative diseases (ND) seriously affect the quality of life and overall life expectancy of patients. Diseases, including Parkinson’s disease (PD), Alzheimer’s disease (AD), Huntington’s disease (HD), and others, refer to nervous system pathological states due to a decrease in the number of neurons or deterioration in the functional state of these cells over time [1,2]. A significant problem is the lack of effective therapeutic approaches to neurodegenerative diseases, often called aging-related disorders, which is largely due to insufficient knowledge of the molecular mechanisms of development of ND, including mitochondrial dysfunction [3].

For example, caloric restriction and an active lifestyle (physical exercise) seem beneficial and crucial in promoting longevity to a certain extent. Still, they are not remedies and will not rescue the lives of patients with already developed neurological disorders [4]. The problem seems immensely relevant, given that, according to statistical forecasts, the total number of people suffering from some form of ND will reach more than 120 million only by 2050 [5].

Neurodegenerative processes have been identified as the main pathophysiological changes in the majority of ND [6]. These diseases can be classified as different types of pathologies, characterized by progressive loss of various neural populations. Symptoms of the diseases can vary depending on the type of nervous system cells that are affected and can range from motor dysfunction to behavioral changes [7].

Despite the variety of manifestations, ND demonstrates the presence of common features, such as the specific protein aggregates or ROS/RNS that ultimately result in neuronal dysfunction and death in time. It is assumed that there is a general mechanism for the development of ND, and mitochondrial dysfunction probably plays a leading role [8]. But this is still an unresolved issue, whether ROS/RNS are the primary cause of the mitochondrial defects or the outcome of accelerating deterioration.

According to modern theories, with almost all ND, researchers observed a weakened energy metabolism of mitochondria [9,10]. Thus, studies on patients have more than once demonstrated a reduced activity of the mitochondrial ETC components, for example, Complex IV in AD, Complex I in PD, and Complexes II and III in HD [11]. There are clinical trials carried out with novel compounds targeting mitochondria as a promising practical approach to treat NDs, particularly while associated with a broad range of mutations mapped in mitochondrial DNA (mtDNA) isolated from the patients [2,12].

It is common knowledge that the tricarboxylic acids cycle (TCA) (i.e., Krebs cycle) produces a significant amount of energy [13] and thus is essential for maintaining a plethora of cell functions. The NADH and FADH_2_ generated in the TCA cycle are further used in the oxidative phosphorylation process to produce ATP [14,15]. Understanding the contribution of TCA cycle enzymes (encoded in the nucleus) and TCA cycle metabolic intermediates in maintaining mitochondrial fitness will help elucidate their contribution to the development of a comprehensive “cause and effect” view in a progressive mitochondrial dysfunction and to the design of new treatments based on TCA cycle enzymes [16].

Aconitase (aconitate hydratase; EC 4.2.1.3) is an evolutionarily conserved enzyme of the TCA cycle. In mammalian cells, the enzyme exists in a cytosolic (Aco1) and mitochondrial (Aco2) isoform, and both isoforms carry out the isomerization of citrate to isocitrate. In addition, the biological role of Aco1 is extended to the maintenance of iron homeostasis, while Aco2 additionally plays an essential role in controlling ATP generation and prevents mitochondrial DNA (mtDNA) instability [17,18,19].

The presence of a covalently bound [4Fe-4S] iron–sulfur cluster (ISC) that is required for catalytic activity is a characteristic feature of both isoforms [20,21]. It has been well documented that ROS/RNS inactivate Aco2, due to its sensitivity to oxidation, making Aco an indicator of oxidative stress and also an indicator of the redox state in mitochondria [22,23].

Considering the moonlighting biological roles performed by this enzyme, as well as the high sensitivity to oxidative stress, it becomes clear why the Aco2 dysfunction in cells can lead to a deficiency in the TCA cycle, ETC functioning, and mtDNA depletion [24]. Intracellular accumulation of oxidants under pathological and age-related conditions has been often found associated with the decrease of Aco activity and posterior exacerbation of free radical formation and oxidative damage of the cells [25,26]. However, the relationship between Aco dysregulation and neuronal metabolic processes requires further investigation.

In this review, we are discussing mitochondrial bioenergetic failure in neurodegeneration with a special focus on the mitochondrial Aco2 and its activity that is regulated by reactive oxygen species and other cellular factors. We present data on biological roles played by aconitase in eukaryotic model organisms that lead to the loss of numerous and diverse functions of mitochondria. We also pinpoint how perturbations in aconitase activity could underlie the pathophysiology of specific neurodegenerative disorders.

Based on the available literature [26,27,28,29,30,31,32,33], the Aco2 enzyme contribution to the ND brings an updated view of the topic.

## 2. Mitochondrial Aconitase Structure and Functions

Even though the mechanisms for crucial neurodegenerative events remain to a large extent unclear, increased generation of ROS/RNS is known to forego or conduct these pathological processes, consistent with the point of view concept that oxidative stress represents the ones of important contributors to ND pathogenesis [34].

As it is known, oxidative inactivation of Aco2 leads to the liberation of redox-active Fe^2+^ and H_2_O_2_, which promotes pathological condition development [35]. It was also shown that lowered Aco2 activity in cells can lead to a deficiency in cellular respiration and mitochondrial DNA exhaustion [24].

These and similar data necessitate a more detailed consideration of the role of Aco2 in physiological conditions and the pathogenesis of diseases.

### 2.1. General Characteristics of the Aconitase

Aconitase (aconitate hydratase; cis-aconitase; AcnB; 2-methylaconitate hydratase; citrate hydrolyase; EC 4.2.1.3) is an iron–sulfur-containing dehydratases and catalyzes the reversible transformation of citrate to isocitrate in the TCA cycle.

**Figure 1:**
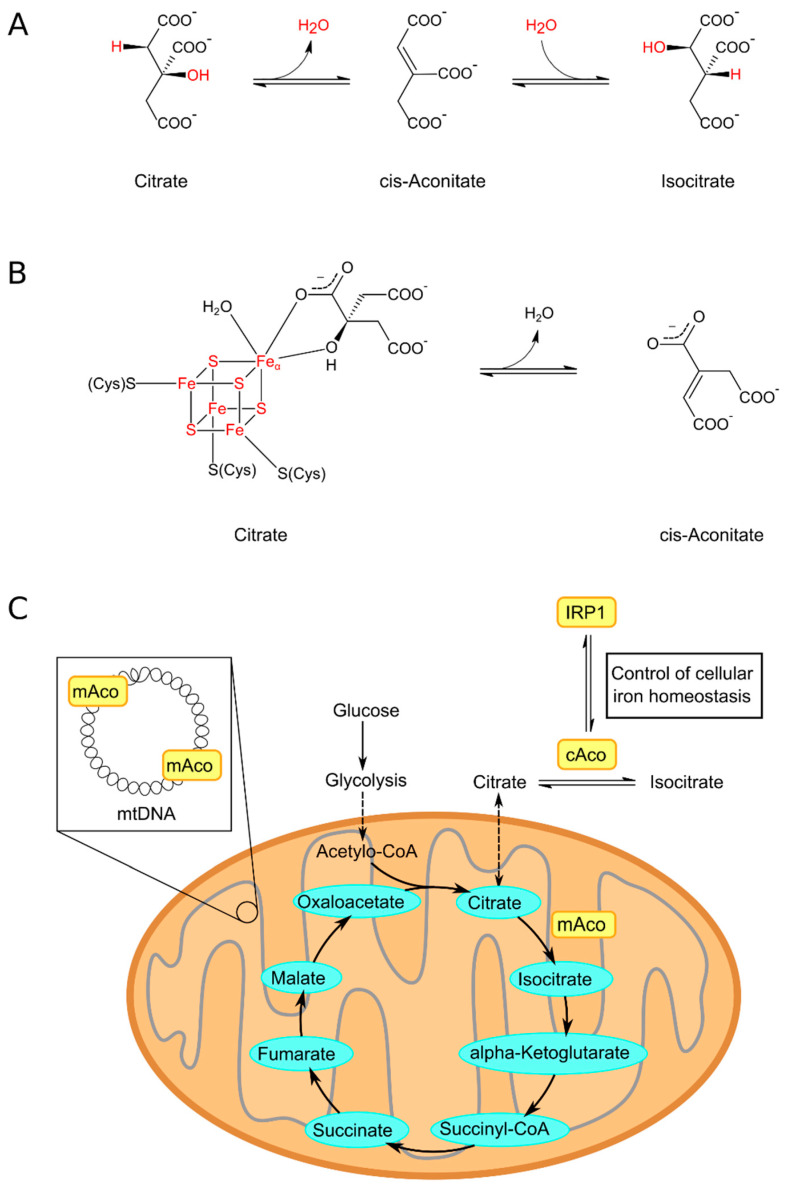
Aconitase engagement in the TCA cycle. (**A**) Aconitase isomerization citrate to isocitrate; (**B**) The mechanism of aconitase reaction. The transformation of citrate to cis-aconitate is promoted by the ISC. The supplement of labile Feα converts an inactive aconitase into its active form and coordinates with oxygen atoms of citrate and water molecules to promote the reaction catalysis; (**C**) Aconitase cellular localization. cAco—cytosolic aconitase (Aco1); mAco—mitochondrial aconitase (Aco2). The redox state of the Aco1 ISC defines the function of the enzyme. IRP-1 is an oxidized (apo-) form of cAco, which takes part in maintaining iron homeostasis in the cell, whereas the reduced (holo-) form—similar to Aco2—accomplishes the isomerization of citrate into isocitrate. Additionally, Aco2 is a protein with a role in the stabilization of mtDNA.

The enzyme, according to the international nomenclature, is a citrate (isocitrate) hydrolyase (cis-aconitate-forming), but usually it is called aconitase (Aco). This is because the official naming does not provide a complete description of the function of this enzyme. After all, Aco is an isomerase, which provides the reaction of transformation of citrate into isocitrate (or in the opposite direction) and hydratase, carrying out the process of formation of cis-aconitate (Figure 1A).

Structurally, the Aco of eukaryotes contains four domains. The three of them are located around the ISC. These clusters are attached to the enzymatic active site by binding three cysteine residues to three iron ions of the cluster. The fourth iron is labile (so-called Feα) and is bound to the OH− group of the substrate and H_2_O. Active enzyme [4Fe-4S]^2+^ cluster facilitates dehydration and rehydration reactions. The Feα of the iron–sulfur cluster is loosely bound and can be released from the ISC, resulting in the formation of an inactive enzyme comprising the [3Fe-4S]^+^ cluster (Figure 1B) [27,36].

It is well known that aconitase is present in organisms of various levels of organization, and two isoforms of the enzyme have been discovered in higher eukaryotes: cytosolic aconitase (Aco1) and mitochondrial aconitase (Aco2) (Figure 1C). However, the main focus of this review will be on the mitochondrial aconitase (Aco2) of higher eukaryotes. In humans, the Aco2 gene (ACO2) is localized on the 22nd chromosome (location: 22q13.2) [37].

A characteristic feature of aconitase is that while most Fe-S-containing proteins function as electron transporters, the enzyme ISC directly interacts with the reaction substrate [38]. Aconitase’s ISC is considered a very important part of the enzyme, playing an essential role in the normal physiology of organisms and offering potential new therapeutic targets for disease treatment (the issue will be discussed in more detail in Section 2.3) [36].

Aconitase structure, thus the enzyme’s proper function, is supported by the mitochondrial HSP70 heat shock protein mortalin. The chaperonin has a role in ISC biogenesis and proper insertion of apoproteins, therefore in the protein maturation [39]. When mitochondrial chaperonin (mtHSP70) is involved in proper folding in the mitochondrial matrix, the cytosolic chaperons of the HSP70 class are engaged in precursor protein transport into mitochondria [40].

It was shown [41] that the first three associated N-terminal aconitase domains are connected with a larger C-terminal fourth domain. It has been reported, that, at least for Aco 1 from *S. cerevisiae* (the homolog of human Aco2 of a similar structural architecture), the N- and C-terminal domains of enzyme interaction are important for modulation of the aconitase posttranslational transport into mitochondria by Ssa1 chaperone (cytosolic Hsp70 class). Ssa1 breaks this interaction and adversely influences the import of the mitochondrial aconitase, as well as its dual localization [41]. Of note, the Ssa1 chaperone, which takes part in the folding of polypeptide chains, displays a distinct activity in aged cells that could contribute to proteostasis collapse during the late stages of aging [42].

Another observation in fission yeast connects Aco2 to the translational process in mitochondria [43]. Aco2+ (SPBP4H10.15) encodes an enzyme domain connected with mitochondrial ribosomal protein L21 (Mrpl49). Aco2-depleted cells of a conditional mutant strain are shown to have diminished synthesis of proteins of respiratory complexes and ATP synthase (Cox/1/2/3, APT6) [43].

### 2.2. The Role of Aconitase in mtDNA Maintenance

Another aspect of understanding the impact of aconitase on the development of neurodegenerative diseases is its role in maintenance of mitochondrial genome stability, which ensures mitochondrial homeostasis. mtDNA instability is one of the biomarkers of compromised mitochondrial function.

Expression of aconitase Aco1p in yeast is controlled by two metabolic regulatory systems: the retrograde system (RTG), which is activated in cells with dysfunctional mitochondria, and the heme activator protein (HAP) system, which is active in cells with normal respiratory activity [44]. When both regulatory pathways are inactivated, the Aco1p level decreases and the number of point mutations and ssDNA breaks in mtDNA increases [45].

Mammalian cells have their intrinsic variant of the central stress-mediating transcription factor, NF-κB. This factor performs more diverse functions, including participation in the induction of apoptosis and immune responses at high levels of mtDNA and nuclear DNA (nDNA) damage induced by ROS. The NF-κB participation in chromosome stability maintaining and mitochondrial respiration regulating may indicate a conserved mechanism also found in yeast [44]. It was shown that some genes encoding the enzymes of the TCA cycle, including SUCLA2, IDH1, IDH3A, and ACO2, were multiply bound by NF-κB [46].

Mitochondrial DNA is particularly sensitive to ROS for several reasons. First, mtDNA is located close to the mitochondrial inner membrane with high levels of ROS liberation from the ETC. In addition, mtDNA is more susceptible to oxidative damage than nDNA because it is not associated with histones, which protect nDNA from ROS [47,48].

Although a lot of mitochondrial proteins are encoded in the nucleus, rRNAs and tRNAs encoded in the mitochondria are also used in the production of mitochondrial proteins, including subunits of the respiratory chain complexes required for ATP making through the oxidative phosphorylation system. Therefore, the production of ROS and the integrity of mtDNA are directly bound to the health and function of mitochondria [49,50].

The mechanisms of regulation of mtDNA expression in response to changes in cell needs are currently being actively studied [51].

It is known that mtDNA exists in association with proteins with roles such as packaging and replication, forming a nucleoprotein complex [52,53,54]. Besides DNA packaging protein, which is necessary for the maintenance and expression of mtDNA and the formation of the basic structure of the nucleoid in mammals [55], it was also established that the nucleoids have proteins whose functions, at first glance, are not related to the functioning of mtDNA [52,54,56].

According to the MitoProteome database, the modern version of Mitoproteome contains information about more than 1700 genes and more than 3600 proteins [57]. These proteins include endonucleases for RNA precursor processing, RNA polymerase, transcription factors, aminoacyl-tRNA synthetases, RNA-modifying enzymes, the structural components and biogenesis factors for the mitochondrial ribosome, translation factors, and other ancillary factors [51,57]. In the context of aconitase association with the mtDNA-nucleoprotein complex, it is assumed that Aco2 is a moonlighting protein with a role, as suggested, in the stabilization of mtDNA by nucleoids remodeling to influence mitochondrial gene expression according to the changing situation in the cell. [19].

Alternatively, aconitase, as the mtDNA-associated protein, may defend mtDNA against ROS. For example, Chen et al. [45] showed that Aco1p defends yeast mtDNA from over-accumulation of ssDNA breaks and point mutations. In addition, they found that the enzyme can repress the reductive recombination of mtDNA. As it is known, a decrease in mtDNA instability in cells lacking the mtDNA packaging factor Abf2p (the homolog of human TFAM) is possible due to overexpression of Aco1p and the ability of the enzyme to directly interact with dsDNA [58]. Like Abf2p, Aco1p also shows preferential binding to dsDNA GC-containing sequences in vitro. Given evidence that Aco1p is required for mtDNA maintenance even in the presence of Abf2p, and Aco1p also interacts with ssDNA, it was hypothesized that Aco1p, but not Abf2p, may defend ssDNA sites [45].

These data support the authors’ [59] view that multifunctional enzymes (such as Aco) have activities that go beyond their generally accepted roles in cell metabolism, including repairing DNA damage, regulating gene expression, and influencing cell homeostasis in general.

The studies by Farooq et al. showed another possible mechanism for mtDNA loss in *aco1Δ* mutant yeast cells [60]. They claimed that the lack of Aco1p in *aco1Δ* activated the RTG pathway, leading to increased expression of citrate synthase and subsequently iron overload in the mitochondria, causing the formation of hydroxyl radicals and oxidative damage to mtDNA. This view is supported by the fact that deletion of RTG1/3 or genes encoding citrate synthase avoided mtDNA instability in *aco1Δ* mutant yeast [60].

As it is known, mutations in the genes coding the Yfh1 protein (the homolog of frataxin in the yeast) lead to iron accumulation in mitochondria, abnormalities in the maturation of ISC-containing proteins (including aconitase), and further mtDNA instability [61,62]. Considering these data, Farooq et al. [60] suggest that suppression of mtDNA instability resulting from mutations in ACO1 and YFH1 by reduced iron and citrate levels indicates that mtDNA destruction in Yfh1 mutant yeasts can be an oblique effect of aconitase dysfunction.

According to Kim et al. [63], overexpression of Aco2 decreased induced by oxidants mtDNA damage and apoptosis in alveolar epithelial cells, while silencing Aco2 by siRNA reversed the changes and enhanced mtDNA damage.

The authors suggest a novel role for mitochondrial 8-oxoguanine DNA glycosylase (a crucial enzyme in the base repair process) and Aco2 in epithelial cells’ mtDNA integrity maintenance oxidative stress conditions. They believe that preventing oxidative destruction of mtDNA by this enzyme chaperoning of aconitase may be an effective way to modulate the cell’s oxidative damage [63].

As is known, the oxidized form of Aco2 in animal cells is first of all degraded by Lon protease (one of the key enzymes in the abnormal protein destruction in the matrix of mitochondria) [64] that, probably not coincidentally, links to mtDNA [65].

G.S. Shadel [19] suggested that nucleoids may be remodeled by Lon through the degradation of mtDNA-bound Aco2, thereby regulating the distribution of the enzyme between the TCA cycle and mtDNA-bound aconitase pools depending on cellular conditions changing.

Altogether, the observations indicate that mitochondrial aconitase may be an important target of cell signaling mechanisms that manage the expression of mitochondrial and nuclear genes, while the mtDNA-related enzyme function provides direct modulation of the expression of genes through interaction with the mitochondrial nucleoid.

It should be noted that a function of Aco2 involving direct interaction with nucleic acid is not unheard of. As it is known, IRP-1 is an apo-form of cAco in animal cells that binds to mRNAs [66] (discussed further in Section 2.4).

In addition, the interaction of aconitase with chromatin has been shown in *Schizosaccharomyces pombe* [67]. In this species, there are Aco1 and Aco2, and both isoforms participate in the mitochondrial TCA cycle. In addition, Aco2 also has nuclear localization. Authors have found that Aco2, by interacting with Chp1 (an HP1 protein that binds to the methylated Lys-9 residue of histone H3), maintains heterochromatin functioning [67].

Moreover, Liu et al. [68] recognized a “non-classical” TCA cycle in isolated mouse liver nuclei (nTCA cycle) and claimed that all enzymes associated with the TCA cycle, including Aco2, are located here also. They showed that the nTCA cycle is directly related to the dynamics of chromatin activity and regulation of transcription. Thus, this study indicates the potential for “non-classical” Aco involvement in intertwining TCA metabolic processes with epigenetic regulation of the nuclear genome [68].

### 2.3. Mitochondrial ROS/RNS Generation as One of the Well-Established Causes of Aconitase Dysfunction

**Figure 2:**
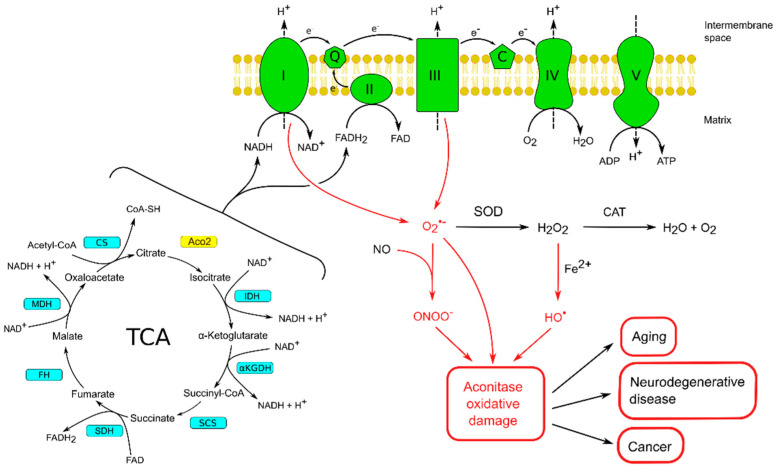
Mitochondrial ROS/RNS generation is believed to act as the main cause of aconitase oxidative damage. One of the main functions of the TCA cycle is the generation of electrons used by the mitochondrial ETC to produce ATP. The multi-subunit complexes of ETC are located in the inner mitochondrial membrane. The electrons provided by NADH and FADH_2_ from the TCA cycle are transmitted to NADH ubiquinone reductase (complex I) or succinate dehydrogenase (complex II), and then to ubiquinol-cytochrome c reductase (complex III), and finally to oxygen through cytochrome c oxidase (complex IV). The functioning of mitochondria is indissolubly connected with ROS production; firstly, superoxide anion (•O_2_−). Superoxide is converted to hydrogen peroxide (H_2_O_2_) by the enzyme superoxide dismutase (SOD) in mitochondria. H_2_O_2_ is then converted to water by catalase (CAT). Superoxide can damage iron–sulfur proteins such as aconitase, subsequently releasing ferrous iron. The presence of ferrous iron promotes the formation of a hydroxyl radical (HO•) from hydrogen peroxide. Superoxide is also capable of reacting with nitric oxide (NO.) to form peroxynitrite (ONOO−). •O_2_−, HO•, and ONOO− can cause extensive oxidative damage to aconitase. Abbreviations: CS, citrate synthase; Aco2, aconitase 2; IDH, isocitrate dehydrogenase; αKGDH, α-ketoglutarate dehydrogenase; SCS, succinyl-CoA synthetase; SDH, succinate dehydrogenase; FH, fumarate hydratase; MDH, malate dehydrogenase.

As it is well known, during the OXPHOS in the mitochondrion, four protein complexes of the ETC move electrons received from NADH or FADH2 produced in the TCA cycle (Figure 2). Electrons moving through the ETC assist the proton–motive force (PMF) creation used to phosphorylate ADP to ATP via ATP synthase [69] (Figure 2).

This process is also associated with the formation of superoxide and hydrogen peroxide, primarily by complexes I and III of the ETC (Figure 2). When produced at a high level and sufficient to overwhelm the antioxidant detoxification ability of the cell, hydrogen peroxide can react with iron in the Fenton reaction with the production of dangerous HO• (Figure 2) [70]. It has been shown that even a short-term exposure to ROS can lead to inactivation of ISCs, which sufficiently stops the energy production [71] and leads to serious consequences for the cell [72].

All these facts force us to look for additional approaches to reducing the level of free radical produced by mitochondria. Such approaches may be antioxidants using or attenuation of free radical production by reducing mitochondrial membrane potential (MtMP).

As it is well known, high MtMP promotes superoxide production [73], while a decrease in membrane potential can lead to a significant decrease in free radical production [74].

This effect can be achieved using small doses of uncouplers, which leads to a moderate decrease in MtMP, which is nevertheless sufficient to reduce the production of free radicals in mitochondria. Several studies in animal models support the validity of the “uncoupling to survive” hypothesis, which suggests that moderate uncoupling of the electron transport system and oxidative phosphorylation, leading to a decrease in mitochondrial ROS production, may be a promising approach to increasing the lifespan of the organism [75,76].

Several studies have reported the beneficial effects of low concentrations of the uncoupler 2,4-dinitrophenol in neurodegenerative disorders [77]. According to Hubbard et al., mild mitochondrial uncoupling offers a promising treatment for traumatic brain injury since uncoupling can reduce oxidative stress and stimulate mitochondrial proteostasis and mitophagy of damaged organelles [78]. These effects confirm the validity of the concept of a significant deposit of mitochondrial ROS production in the pathogenesis of ND.

As noted earlier, Aco2 has been proposed as a redox indicator in the mitochondria [79], and it is one of the protein candidates for oxidative inactivation. Tretter and Adam-Vizi provide evidence that aconitase is the most sensitive enzyme in the TCA cycle to the inhibitory effect of hydrogen peroxide [80].

It is important that in some ND in the development of which oxidative stress is involved, as well as in different models of these types of disorders, a decrease in aconitase activity has been repeatedly demonstrated [35].

**Figure 3:**
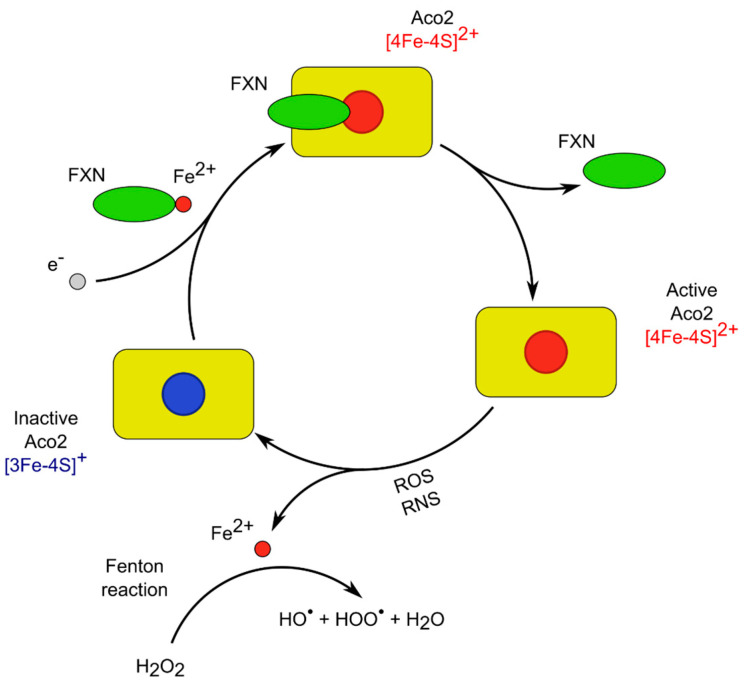
Maintenance of the balance between an active and an inactive Aco2. The active form of the enzyme (ISC [4Fe-4S]^2+^) can be oxidized by free radicals to the inactive form (ISC [3Fe-4S]^+^). With the participation of Fe^2+^ in the Fenton reaction, free hydroxyl radicals are generated, which additionally oxidize mitochondrial macromolecules. Frataxin (FXN), due to its participation in the biosynthesis of ISCs, promotes the restoration of the active form of Aco2 in the presence of reducing agents, such as glutathione or ascorbic acid. Adapted with changes from [38] with the permission of the authors.

It has been reported that exposing purified Aco1 or Aco2 to hydrogen peroxide or superoxide results in the release of Feα from the iron–sulfur cluster and enzyme inactivation (Figure 3). For example, Bulteau et al. provided data that when mitochondria were treated with hydrogen peroxide, inactivation of aconitase was observed, and EPR spectroscopic analysis data indicated that Feα release from the ISC preceded enzyme inactivation [81]. According to the authors, long-term exposure of mitochondria to stationary levels of superoxide or hydrogen peroxide led not only to the destruction of the iron–sulfur clusters but also to carbonylation and degradation of the enzyme [81].

As it is known, •O_2_− is the prevalent form of free radicals generated by mitochondria [70] capable of reacting with nitric oxide (NO•) to form peroxynitrite (ONOO− (Figure 2). It was shown [82] that ONOO− promotes the transformation of the ISC of pig heart aconitase into the inactive form with the loss of labile Feα. It was shown that the interaction of peroxynitrite with aconitase led to the nitration of tyrosines 151 and 472 and the oxidation of cysteines 126 and 385. Significantly, cysteine 385 was associated with the ISC, while other modified cysteine and tyrosine residues were localized near the binding site. This data gave the authors reason to assume that these modifications cause conformational changes leading to disruption of the active center. Thus, ONOO− not only disrupts the iron–sulfur cluster but can also alter aconitase activity through modifications of enzyme amino acids [82].

As noted above, aconitase is not only sensitive to the action of free radicals but can itself participate in their formation [79]. Therefore, this enzyme can be both a “victim” of oxidative stress and its initiator [83].

In general, during oxidation, the [4Fe-4S]^2+^ cluster group loses the Feα, which leads to the formation of an inactive Aco containing [3Fe-4S]^+^. The released in this process Fe^2+^ takes part in the generation of free hydroxyl radicals, which can actively oxidize mitochondrial macromolecules [35,84,85]. The inactive form of the Aco2 can be reactivated by restoration of the ISC by Fe^2+^ in the availability of reducing agents (such as ascorbic acid or glutathione). Frataxin is also engaged in the reconstruction of the oxidatively inactivated ISC [38,86] (Figure 3).

Frataxin fulfills a facilitator’s role as an activator of L-Cys desulfurase NFS1 supercomplex for the iron–sulfur cluster mounting [87,88] and also as a chaperone having several centers for metal ions binding [38,89]. 

It was postulated that FXN interacts with Aco and decreases the intensity of oxidant-induced enzyme inactivation [86]. It was shown that frataxin facilitates and stabilizes the transfer of the Fe^2+^ to aconitase to convert it into its active form [90,91]. According to Cherif et al., the aconitase activity was positively modulated by the frataxin level in mitochondria and increased in vitro and in vivo by the increased frataxin expression [92]. As is known, mutations of Yfh1 (the yeast homolog of FXN), bring iron accumulation in mitochondria, defects in the aconitase formation, and mtDNA instability [62,93].

The above data supports the view that Aco2 acts as a “redox rheostat” (similar to an electrical device used to control current by changing resistance), adjusting the level of superoxide production in TCA, as well as correlating the direction of metabolic processes with the actual energy or anabolic needs of the cell. The latter function is achieved by “redirecting” citrate to produce NADH or into the acetylation reactions or fat synthesis in the cytosol [94]. It is assumed that blocking the TCA cycle due to dysfunction of aconitase contributes to a reduction in the efficiency of the mitochondrial ETC [94]. Thus, the reaction catalyzed by Aco2 might be one of the centers of regulation of ROS formation.

The immensely high sensitivity of the Aco2 to ROS made it possible for researchers to use the level of enzyme activity as an indicator of free radical production in intact cells or isolated mitochondria [95], as well as an indicator of cells oxidative damage [96].

Moreover, aconitase dysfunction has been seen in different models of diseases in the pathogenesis of which free radical accumulation plays a leading role [35]. Along with the participation of aconitase in the development of neurodegenerative diseases [27] (in some cases age-dependent), its biological role and contribution to the actual mechanisms of aging are quite widely discussed in the literature.

Aco2 was found to be one of the major oxidant targets during senescence or pathological conditions in which the presence of mitochondrial insufficiency is indicated. It is supposed that a decrease in Aco2 activity can make a significant contribution to slowing down the functioning of the TCA cycle and electron transport through the ETC with reduced ATP making. The latter phenomenon causes a decrease in the functional capabilities of the body in old age [97].

Data coming from several studies show that oxidative damage of biomolecules during aging is a specific phenomenon and may be a mechanism by which oxidative stress promotes age-related impairment of certain metabolic functions of the body (reviewed in [98]). The validity of these ideas is confirmed by the identification of the aconitase as a goal of aging-associated oxidative lesion, which makes it possible to estimate the physiological age of a particular organism by measuring the activity of this enzyme and also evaluate the effectiveness of approaches aimed at slowing down the aging process.

### 2.4. Mechanisms of Aconitase Activity Regulation by Iron

Iron is an important chemical element that takes part in the body’s basic metabolic processes as a co-factor for many iron-containing proteins [99,100]. In addition to protein-embedded iron, a small but important part of the iron pool forms in cytosol complexes with low molecular weight compounds like citrate or phosphate. This form of existence of iron is known as a cytosolic “labile (free) iron pool” [101]. The labile iron pool is significant for normal cell functioning, and its dysregulation could be accompanied by cellular dysfunction and the development of pathological conditions involving mitochondrial failure [100]. That is why living organisms have evolved a system of complex regulatory mechanisms to maintain and regulate an optimal pool of labile iron. The dynamic balance between Aco1 and IRP1 is one of the important ways of such regulation [102,103].

The aconitase apo-form, named iron response protein 1 (IRP1), can join the hairpin mRNA sequence, the iron regulating element (IRE). IRE is highly conserved and present in the mRNA of genes involved in the regulation of iron levels in the cell, such as the transferrin receptor (TfR), the ferritin, and the δ-aminolevulinic acid synthase (ALAS), as well as Aco2 [104,105,106]. This binding depends on the level of iron in the cell and contributes to the implementation of post-transcriptional regulation of these genes [103,107].

**Figure 4:**
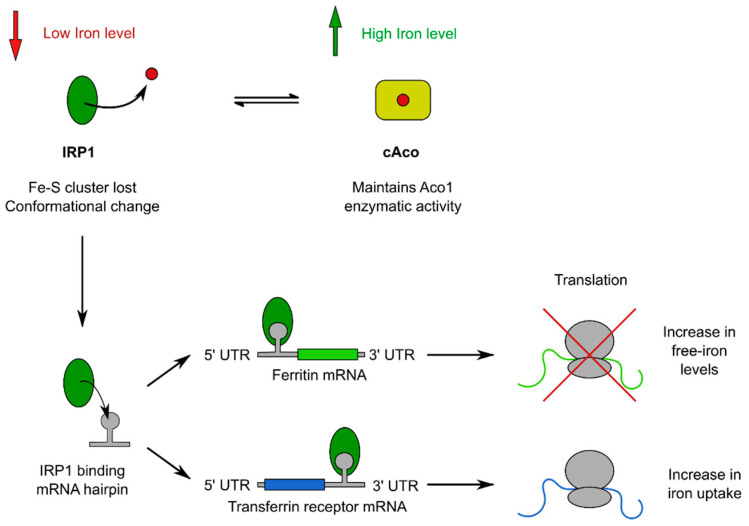
Mechanism of the cAco/IRP1 balance and the binding of IRP1 with IRE mRNA. When the level of iron in the cell is high, IRP1 principally exists in the reduced state with a complete ISC, which allows the protein to carry out its enzymatic function. When the level of iron in the cell is low, the interaction of IRP1 with the IRE located in the 5′ UTR of ferritin mRNA guides to inhibition of the translation of these mRNA, and down-expression of ferritin and ultimately to the increase in the free-iron level. At the same time, the binding of IRP1 on the IRE located in the 3′ UTR of the mRNA encoding the transferrin receptor guides the stabilization of this mRNA and, consequently, an increase in its translation, which allows for the intensification of the process of iron uptake.

It was shown that the connection of IRP1 to the IRE situated at the 5′ UTR of ferritin, ALAS, and Aco2 mRNAs guides to suppression of protein translation and, as a result, an increase in the level of the iron pool in the cell [108,109]. For example, genes important for iron storage and export (such as ferritins and ferroportin) include 5′ UTR IRE, and at a low level of the iron pool in the cell, their activity decreases. This inhibition interferes with iron export when cells are iron deficient, increasing the intracellular pool of available iron [109] (Figure 4).

It is known that translational repression of Aco2 and ALAS synthesis is weakly dependent on iron levels, based on which the authors of the study suggested that this phenomenon may serve for fine regulation of iron-dependent biosynthetic processes [110]. At the same time, it has been shown that the interaction of IRP1 with IRE present in the 3′ UTR of transferrin receptor mRNA guides to its stabilization and hence an increase in its translation, which leads to a growth in iron absorption [103] (Figure 4). In the availability of a high iron level, IRP1 is mainly present in a reduced state with a complete iron–sulfur cluster, functioning as an aconitase [111,112].

As it has been highlighted in Section 2.1, aconitase possesses four functional domains. IRP1 has a hinge region dividing the domains 1–3 and 4 [113,114]. It was assumed that when the iron pool is depleted and the iron–sulfur cluster is lost, the junction region is capable of bending, which allows the protein to acquire a closed or open conformation. When studying the role of ISC in the regulation of the transformation of the open form (IRP1) of the protein into the closed form (corresponding to cAco), it was found that the specific domain interactions, with the help of the ISC, favor the closed protein conformation. At the same time, the open conformation is much more stable in the absence of the stabilization network caused by the presence of ISC [103].

As is known, mammals differ significantly from yeast or bacteria in their mechanisms for maintaining optimal cellular iron levels. While yeast regulates the levels of specific proteins associated with maintaining iron homeostasis at the transcriptional level via the “iron regulon” [115], mammals use posttranscriptional control of the expression of a variety of proteins involved in these processes [116]. As noted earlier, a characteristic feature of genes that maintain iron homeostasis in mammals is the presence of IRE stem loops in their mRNAs. The distribution of IREs along the 5′ or 3′ UTR of certain transcripts is of critical functional importance because when the IRP1 interacts with the 5′ UTR, the translation is absent, and when it interacts with the 3′ UTR, the translation is in progress [103] (Figure 4).

In addition to the iron level, the presence of other factors that can transform Aco1 activity has been shown. For example, data reported by Brown et al. [117] indicate that phosphorylation of IRP1, by affecting the stability and turnover of ISC, may modulate its reaction on the iron level alterations in the cell. It is known that some ROS can modulate IRP1 functions also. Sureda et al. showed that superoxide inactivates IRP1 binding with mRNA through direct chemical interaction, whereas extracellular H_2_O_2_ through a signal system significantly activates IRP1 [118]. According to the authors, this mechanism may contribute to the progress of anemia or inflammatory processes in tissues [118,119].

Thus, the aforementioned data indicate that the functioning of aconitase and iron homeostasis are closely related in animal cells. It can be assumed that the factors that change the activity of aconitase can also affect the fate of iron in the cell, which in turn can lead to the development of pathological processes in the human body. For example, Juang et al. indicated that the expression of Aco2 from human PC-3 cells is activated by iron compounds [120].

## 3. Overview of the Mitochondrial Aconitase Capacity in Neurodegenerative Conditions

Neurodegenerative diseases are conditions that quietly destroy parts of the human nervous system, primarily the brain. These conditions usually develop slowly, and the effects and symptoms tend to appear later in life. The brain is an organ with high metabolic activity, therefore susceptible to bioenergetic failure [121,122].

Mitochondrial aconitase is an important enzyme that connects the TCA cycle and other metabolic systems, is engaged in the regulation of the intracellular iron pool, and being a source of free radical oxygen species, is itself very sensitive to oxidative stress. The scientific literature indicates that aconitase dysfunction is a significant factor in the progress of neurodegenerative processes [27,35,38]. However, its specific role in different kinds of neurodegenerative pathological conditions development remains largely underappreciated.

### 3.1. Parkinson’s Disease

Parkinson’s disease (PD) is one of the most general progressive NDs, affecting over 0.5–1% of those 65–69 years of age [123].

Several risk factors for the development of PD are known, among which are patients’ age [124], smoking, environmental toxins, and even excessive caffeine intake [125]. Although the precise molecular mechanism of the disease remains to be discovered, the primary cause seems to be a progressing metabolic dysfunction in aging cells.

PD is characterized by both motor functional disorders (rigidity, tremor, and bradykinesia) and non-motor manifestations (depression, apathy, cognitive dysfunction, fatigue, and dementia) [126].

Atrophy of the frontal cortex and enlargement of the ventricles are often found in patients with PD, but the most characteristic morphological change is the death of dopaminergic neurons in the brain locus coeruleus and substantia nigra pars compacta [127].

**Figure 5:**
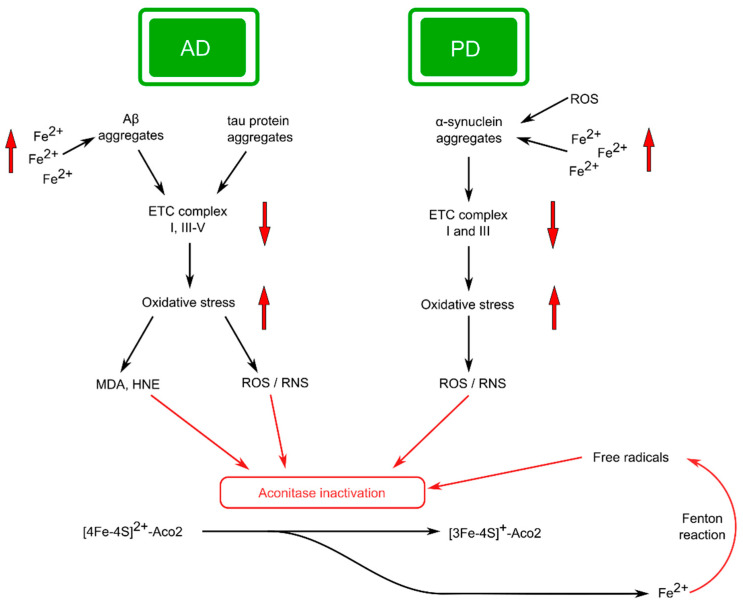
Schematic figure illustrating some of Aco2 affecting pathways in AD and PD. Alzheimer’s disease (AD): Aggregation of Aβ plaques and tau tangles intensifies with increasing iron concentration. Aβ and tau protein aggregates interact with ETC complexes I, III–V and disrupt their function leading to overproduction of free radicals (where complex I exhibits maximum activity). •O_2_− and ONOO− can directly modulate the activity of aconitase. The products of lipid peroxidation (MDA and HNE) also modify enzyme activity at the post-translational level. Parkinson’s disease (PD): Activation of α-synuclein by ROS leads to cytoplasmic protein aggregation. Fe^2+^ accumulation stimulates increased α-synuclein synthesis and aggregation. These aggregates cause inhibition of ETC complexes I and III. Dysfunction of ETC enzyme complexes in dopaminergic neurons of the SN contributes to the inactivation of Aco2 by intensifying the production of ROS in the patient’s brain. In both AD and PD, an active form of the enzyme (ISC [4Fe-4S]^2+^) can be oxidized by different forms of free radicals, forming the inactive Aco2 form (ISC [3Fe-4S]^+^) and releasing Fe^2+^. Fe^2+^ promotes the generation of hydroxyl radicals in the Fenton reaction, with the subsequent Aco2 oxidation. Abbreviations: Aβ, amyloid-beta; HNE, 4-hydroxy-2-nonenal; MDA, malondialdehyde; ROS, reactive oxygen species; RNS: reactive nitrogen species.

Microscopically, neurons in PD are characterized by the availability of Lewy bodies (protein aggregates formed primarily by alpha-synuclein) and abnormal neurites (Lewy neurites) [124,128] (Figure 5). Protein misfolding also occurs in PD [128].

Mitochondrial dysfunction and depressed bioenergetic processes are currently considered to be one of the preferred mechanisms of PD pathogenesis [129]. This becomes clear if we take into account the fact that dopaminergic neurons, due to the presence of long and branched axons, a large number of intercellular contacts, and active generation of action potential, take significantly more energy compared to other types of neurons [130]. For example, according to authors [131], mitochondrial impairment occurs with Lewy body formation. It was shown that loss-of-function mutations in PARK7 (DJ-1) caused disruptions in the assembly of complex I and the functioning of OXPHOS in general, which naturally led to decreased ATP production and oxidative stress while increasing glycolytic activity [132]. These data suggest that mitochondrial malfunction may be a cause of inadequate protein aggregation [133]. Zhu et al. [28] found that aconitase deficiency increases the organism’s likelihood of developing PD by promoting mitochondrial malfunction and stopping autophagy by inactivating the transcription of genes associated with this process. According to the authors, these data support the Aco2-mediated way for the formation of PD. However, the connection between Aco2 and PD has not been sufficiently clarified [28]. As noted earlier (Section 2.3), NO· may react directly with Aco2, which leads to enzyme dysfunction. The adverse effects of nitric oxide on aconitase may be due to the active derivatives formed, such as ONOO− or S-nitrosothiols [134]. Investigations of the brain of patients with PD and animal model studies suggest that nitric oxide may play an important role in the mechanisms of development of PD [135].

It was shown that reduction of ETC complex I activity has a substantial effect on Aco2 functioning, significantly decreasing Aco2 activity in time in a concentration-dependent manner [136]. Accordingly, it was assumed that complex I dysfunction leads to the overproduction of ROS, which damages mtDNA and other oxidation-sensitive elements of mitochondria, including Aco2, as reported by Celardo et al. [29]. The study [136] provides evidence that dysfunction of complex I of the mitochondrial respiratory chain has an essential influence on the content of iron–sulfur clusters and, accordingly, the activity of enzymes containing ISC, including Aco2 (Figure 5).

It is worth emphasizing that in the dopaminergic neurons, oxidative stress leads to the formation of both ROS and dopamine quinone and inactivation of the mitochondrial and cytosolic aconitases. The role of dopamine quinone in the covalent modifications of aconitase in these neurons, according to Yoon et al. [137], may be the factor that makes these cells especially defenseless.

As it is known, the Aco2 enzymatic activity is regulated by many factors, but primarily these factors involve ACO2 gene variations, post-translational aconitase alterations, and ROS attack on iron–sulfur clusters [28,138].

In this line, data obtained by authors [139] indicate an essential intensification of the carbonyl modification of Aco2 in the brain substantia nigra (SN). Oikawa et al. speculated that Aco2 carbonylation not only disrupts the synthesis of ATP in the cells but may also be the reason for the intense release of iron in the SN [139].

Iron accumulation in the SN has been reported in patients with PD [140]. Currently, the widespread point of view is that the accumulation of iron in PD contributes to the excess production of ROS, which in turn leads to the oxidation of proteins, DNA, and other biomacromolecules. The result of these reactions can be significant structural and functional failure in the SN [141] (Figure 5).

Mena et al. showed that inhibition of ETC complex I leads to a reduction in the synthesis of iron–sulfur clusters and an increase in the intensity of IRP1 interaction with mRNA, and these processes are accompanied by an enlargement in the cytosolic pool of “free iron” [136] (Section 2.4). Research results that an inhibition of ETC complex I leads to an increase in IRP1 activity are compatible with early studies showing an increase in IRP1 activity in tissues of patients with PD [142]. Overall, these data may indicate that IRP1 activation is a link between complex I inhibition and Fe^2+^ accumulation, which are characteristic features of idiopathic PD [143].

Intense production of ROS by mitochondria, leading to increased IRP1 activity, could, in theory, intensify the translation of α-synuclein as an adaptive cellular reaction [141]. These data may indicate a close connection between iron metabolism and α-synuclein accumulation in the development of PD (Figure 5). It can probably be assumed that this kind of molecular cooperation can lead to the formation of a “vicious” cycle in which iron stimulates the accumulation of α-synuclein, which leads to increased aggregation and transmission of this protein, which in turn contributes to the progression of PD. The effect can be intensified by mitochondrial ROS production [141]. Estimation of iron levels in the brain of a patient with PD may possibly be used to assess the development dynamics of the disease in the clinic [141].

Studies of patients with PD have shown not only the elevated vulnerability of their neurons to changes in mitochondrial energy production but also established that the level of iron in the PD brain is significantly higher than in the control group of the same age [144,145]. It has also been shown that Aco2 activity reduction can promote significant changes in iron homeostasis, and high levels of iron in the SN may contribute to the progression of PD.

Significantly, changes in the regulation of iron homeostasis were also observed in other brain regions of patients with PD. According to Yu et al., iron content in the temporal cortex was significantly lower, and levels of iron-related proteins were also essentially reduced in this part of the brain [146].

It needs to be mentioned that, in the report by Liu et al. [147], baicalein, an antioxidant flavonoid from the *Scutellaria baicalensis* roots, has the potential to connect with Aco1 and to defend this enzyme from the induced by free radicals oxidative stress. Moreover, baicalein promotes aconitase activity and inhibits IRP1 activation as a transcriptional regulator, in the PD animal model. In addition, this flavonoid decreases the level of iron-produced hydroxyl radicals. The authors conclude that the baicalein reduces iron accumulation and iron-dependent oxidative stress in the brain of experimental animals by defending Aco1 [147].

Therefore, high mitochondrial iron levels may contribute to the development of oxidative stress (Figure 3). An interesting study by Esposito et al. shows that oxidative inactivation of Aco2 induces oxidative stress, mediated by iron overload. This stress resulted in the increased membrane permeability and swelling in *Drosophila* PINK1 mutant mitochondria [148]. As is well known, the PTEN-induced kinase 1 (PINK1) is one of the mitochondrial kinases, and the animals carrying Pink1 mutation is considered an experimental model of PD in humans. PINK 1 in combination with PARKIN is involved in the elimination of dysfunctional mitochondria through mitophagy [149]. It was concluded that Aco2 dysfunction and iron accumulation participate in the pathophysiological processes associated with PINK-1 dysfunction.

When analyzing these data, it should be kept in mind that comprehensive studies have shown that PARKIN takes part in many mechanisms of mitochondrial quality control, including their biogenesis and elimination of damaged organelles (mitophagy) [150]. However, these results are largely ambiguous due to conflicting data. Filograna et al. recently demonstrated that the lack of Parkin in mice did not significantly affect the natural decline in mitochondrial function in energy-consuming organs. In addition, it has been shown that the PARKIN deficiency did not reduce OXPHOS activity or provoke mitochondrial dysfunction in the skeletal tissue of a patient with PD [150].

Hence, Filograna et al.’s data do not confirm the participation of PARKIN in mitochondrial quality control and may indicate the availability of PINK1/PARKIN-independent pathways performing the function of maintaining a delicate balance among the formation and destruction of mitochondria [150].

To sum up, the data accumulated suggests that the functional activity of Aco2 is essentially reduced in patients with PD, and the severity of this phenomenon depends on the patient’s age at the beginning of the disease and its duration. Furthermore, the Aco2 inactivation and iron accumulation are involved in PD pathophysiological mechanisms by activating brain tissue oxidative damage (Figure 5). As a result of these processes, free radicals and dysfunctional molecules accumulate in SN neurons, which in turn contributes to cell death. These data not only confirm the important role of Aco2 in the pathogenesis of PD but may indicate that improving energy metabolism by targeting Aco2 can be considered a promising strategy for the treatment of PD and, possibly, other NDs [28].

### 3.2. Alzheimer’s Disease

A set of symptoms that include problems with memory, language, and logical thinking ability is called dementia. About 80% of dementia cases are caused by Alzheimer’s disease (AD) [151]. More importantly, current statistics indicate that the incidence of AD is increasing worldwide, and by 2050, 1 in 85 people in the world will be affected by this disease, with about 40% of patients requiring specialized high-level care [152,153].

The characteristic feature of this disease is the deterioration of cognitive functions due to the progressive death of certain neuronal populations of the brain [154]. Most often, selective memory impairment is observed in the early stages of AD. Impairments in executive function and problem-solving are also clinical demonstrations of the disease [155].

It has been established that numerous factors can contribute to the development of AD. These may be biological (aging, weight, gender, etc.), environmental (toxins, traumatic brain injury, *Porphiromonas gingivalis* infection, etc.), and genetic factors (mutation of the APP gene) [156,157]. Although research to date has significantly improved our knowledge of AD, the basal mechanisms of disease pathogenesis remain largely unclear.

Mutations in the APP or PS (presenilin) genes, which guide the excess production and accumulation of amyloid beta (Aβ), determine the development of familial AD. One of the main features of the AD prognostic phenotype is considered to be the accumulation of Aβ plaques and tau protein tangles, deficiency of acetylcholine, etc. [158,159]. These ideas were formulated in the form of an “amyloid cascade hypothesis,” where aggregation of Aβ may trigger oxidative stress, tau pathology, and a number of other metabolic changes that lead to neuron death and disease development [160] (Figure 5). But these ideas cannot completely elucidate the mechanism of the development of sporadic forms of the disease, which make up more than 90% of dementia cases due to AD [133].

There are also ideas that the microtubule-associated tau protein can be intensively phosphorylated, which leads to the destruction of microtubules, and the released tau undergoes aggregation. These processes naturally lead to disturbances in the axonal transport of mitochondria, dysfunction of synapses, and ultimately the death of neurons [133,161] (Figure 5).

At the same time, some studies indicate that Aβ and tau proteins are most likely not the main cause of AD, and another possible mechanism, primarily connected with mitochondria, has been proposed [162]. Currently, among the pathways through which Aβ causes its pathogenic effects, a significant body of data supports the role of oxidative stress in this process [163] (Figure 5).

For example, studies in a transgenic mouse model of AD suggest that oxidative injury forerun the formation of amyloid plaques, and changes in mitochondrial proteome occur even before [164]. Thus, the important role of mitochondrial abnormalities in the pathogenesis of AD is currently estimated, which eventually led to the proposed “mitochondrial cascade hypothesis of AD” [162].

A significant amount of the data indicate a disruption in the bioenergetic function of mitochondria in AD [154]. Additionally, it was shown that dysfunction of protein complexes involved in oxidative phosphorylation endows to the clinical display of AD, with dysfunction of complex I likely playing a leading role [162,165] (Figure 5).

Awareness of the significance of mitochondrial dysfunction in AD pathogenesis has become the basis for intensifying research into effective treatment strategies aimed at normalizing mitochondrial functions [162]. The main efforts were devoted to finding ways of reducing the intensity of oxidative stress using antioxidant therapy, the target of which was mitochondria. A fairly large number of studies have been conducted on rodent models that have shown the effectiveness of this approach to solving the problem [166]. In this regard, the data that such intramitochondrial antioxidants, like mitoquinone mesylate (MitoQ), astaxanthin, mito-apocynin, and others, demonstrated positive effects in experiments are quite optimistic [162].

As it is known, one of the proposed therapeutic strategies targeting mitochondria directly focuses on modulating mitochondrial bioenergetics. It is believed that Aco2 is one of the main targets of oxidative stress, which is characteristic of AD. It has been established that directly after the formation of amyloid plaques, one of the first mitochondrial enzymes that undergoes inactivation under these conditions is aconitase [30].

Oxidative-sensitive proteins such as Aco2 could also serve as markers for early disease detection and targets for potential therapeutic intervention in AD.

It was shown that aconitase activity can be significantly altered by by-products of lipid peroxidation. For example, 4-hydroxy-2-nonenal (HNE) modified and carbonylated aconitase was found in the cortex of patients with AD [167]. Yarian et al. have shown that post-translational oxidative modification of the Aco2 by malondialdehyde (MDA) may be responsible for an essential decrease in enzyme activity in mouse heart cells [168] (Figure 5).

According to Longo et al. [31], when rat PC12 cells or human neuroblastoma SK-N-SH cells were treated with a soluble or fibrillar form of Aβ1-42 (Aβ-derived peptide), significant inactivation of aconitase was observed with subsequent changes in cell function and loss of their viability. The authors suggested that the intensification of superoxide formation and the liberation of iron from ISC may be the onset of the neurotoxicity of Aβ1-42 [31].

It was shown that a pronounced decrease in aconitase activity in peripheral lymphocytes in patients with AD correlates with sensitivity to oxidative injuries that occur at the beginning of the disease [169].

In addition, the increased accumulation of superoxide anions and their interaction with nitric oxide, the production of which is changed in AD, leads to the formation of extremely reactive peroxynitrite anions [170]. These anions significantly reduce Aco2 activity in the brains of patients with AD [27].

The literature provides data that iron accumulation in the tissues of patients with AD causes lipid peroxidation and oxidative stress by impairing mitochondrial functioning and promoting the organelle’s generation of free radicals (reviewed in [171]).

Evidence from several researchers proposes that disruption of iron homeostasis is one of the first events in AD development. Peters et al. [172] believe that impaired regulation of iron levels and Aβ pathology are synergistic in the neurodegenerative progress and eventually initiate several processes that lead to the display of AD (Figure 5).

It was shown in in vitro experiments that enlargement of the concentration of iron increases Aβ plaque and tau tangle aggregation and their negative influence [173]. In vivo studies have shown that through activation of glycogen synthase kinase 3β and cyclin-dependent kinase 5, iron may be engaged in the intensive phosphorylation of tau protein [174].

It is known that IREs are involved in the control of cellular iron homeostasis by regulating translation or increasing the stability of the mRNAs of specific proteins (Section 2.4). Rogers et al. [175] demonstrated the existence of a novel IRE type II in the 5′-UTR of the APP transcript. According to the authors, the regulation of amyloid precursor protein mRNA by iron indicates the role of iron in this protein metabolism. The study results also indicate that the structure of this RNA may become an informative parameter in the search for promising drugs, such as desferrioxamine (an iron chelator), which reduces Aβ peptide burden during AD.

Taken together, these and a lot of other data indicate that Aβ and tau protein are connected with iron metabolism in the brain, and iron accumulation may cause decreased cognitive function in patients with AD [176]. Based on this information, some researchers suppose that iron accumulation should be embedded in the “Amyloid cascade hypothesis” of AD [177].

As is known, “Ferroptosis” is characterized as a special form of cell death, controlled by iron-related accumulation of lipid hydroperoxides, and the implication of ferroptosis in neurodegenerative diseases has now been widely received. Authors [178] suggested the “ferroptosis hypothesis” in AD, according to which this phenomenon plays a significant role in generating destruction of neurons and cognitive disorders. Accordingly, regulating neuronal iron homeostasis and decreasing ferroptosis may be a promising direction in the treatment of patients with AD [176,179].

Significantly, transition-metal ions, such as copper or silver ions, are also able to interact with the monomeric Aβ with the formation of a metal-bound Aβ complex [180].

Investigating the potential influence of iron metabolism on the hazard of AD progress, Crespo et al. performed patients’ genetic analyses with the estimation of the iron level in peripheral blood by biochemical studies [181]. The results display an essential reduction in serum iron, transferrin, and ferritin levels in patients with AD. Essential connections with AD were detected for single nucleotide polymorphisms in TFR2, SLC40A1, and ACO1 genes, responsible for transferrin receptor 2, ferroportin, and aconitase synthesis, respectively. The authors hypothesize that, shown in the study, AD-related single nucleotide polymorphisms in genes could play a significant role in the dysregulation of genes associated with iron metabolism, which significantly influences the phenotypic manifestation of the disease [181].

To sum up, according to the modern point of view, recovering mitochondrial optimal condition may be an effective approach to AD treatment. The above analysis of data indicates that aconitase activity is diminished in samples of patients’ tissues and correlates with oxidative stress intensity. Additional research is required to confirm the essential role of Aco2 in the pathogenesis of the disease and its significance as an indicator of the dynamics of AD development [162].

### 3.3. Huntington’s Disease

Huntington’s disease (HD) is an ND that affects approximately 15 people per 100,000 in the population [182].

**Figure 6:**
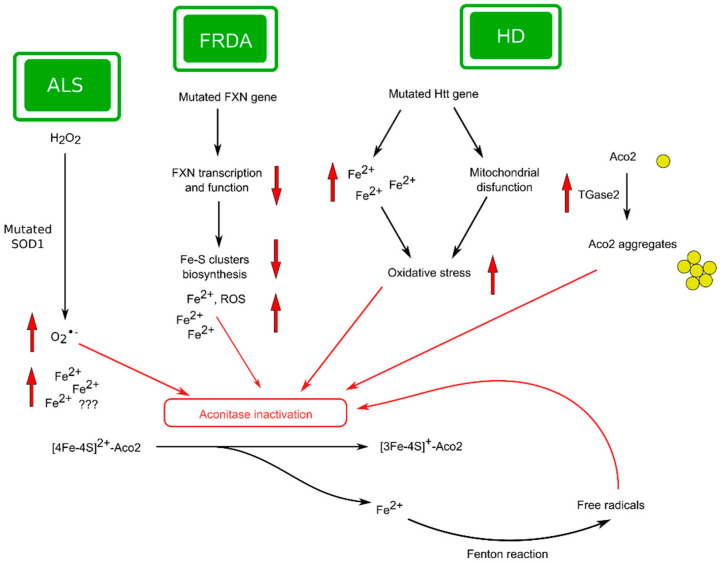
Schematic figure illustrating some of Aco2 affecting pathways in FRDA, HD, and ALS: Friedreich’s ataxia (FRDA): The frataxin function is closely connected to the synthesis of ISC. A mutant FXN gene in FRDA patients leads to functional deficiency of frataxin, iron accumulation, and oxidative stress intensification. Mutated frataxin is responsible for the deficiency of ISC-dependent enzymes in the mitochondria of patients with FRDA. Huntington’s disease (HD): Mutated huntingtin promotes Aco2 dysfunction with the help of TGase 2 through aconitase aggregation into inactive polymers. In addition, the accumulation of iron in the basal ganglia of the brain in the early stages of HD development is considered to be a marker of later iron-induced oxidative stress, leading in turn to changes in aconitase structure. Amyotrophic lateral sclerosis (ALS): Mutated SOD1 produces superoxide anions from hydrogen peroxide, leading to inactivation of Aco2 in ALS patients. The probable role of dysregulated iron homeostasis in ALS pathogenesis requires further study. In all variants of diseases, an active form of the enzyme (ISC [4Fe-4S]^2+^) can be oxidized by free radicals, forming the inactive Aco2 form (ISC [3Fe-4S]^+^) and Fe^2+^ releasing. The elevated levels of Fe^2+^ lead to excessive oxidative damage, which contributes to further oxidative inactivation of aconitase and oxidative stress conditions in neuronal cells. Abbreviations: FXN, frataxin; Htt, huntingtin protein; ROS, reactive oxygen species; TGase 2, transglutaminase 2.

It is a disease characterized by the abnormal propagation of the CAG triplet repeats in the polyglutamine area of the huntingtin (HTT) gene [183]. Huntingtin is a protein that performs several important functions, such as regulation of nervous system development in embryogenesis, taking part in the correct organization of synapses, and others [184]. For example, the presence of HTT at the stage of embryonic development is vital since animals with the HTT gene knocked out do not survive until birth [185]. It is generally accepted that mutation in the HTT gene triggers a lot of molecular processes ending in transcriptional disorders and mitochondrial injury [186] (Figure 6).

Clinically, HD is characterized by progressive cognitive and motor malfunction, caused by deprivation of γ-aminobutyric acid in the striatum spiny neurons [187].

Studies of tissues in patients with HD tissue exhibited a reduction in the activity of mitochondrial complex II and III with a slight decrease in complex IV activity [188]. According to authors [189], inherited complex II faults are bound with basal ganglia degeneration. It has also been shown that in the mitochondria of lymphoblasts in patients with HD, there is enlarged organelle dysfunction, which correlates with the number of CAG repeats [190].

Given the significant functions that mitochondria perform in maintaining neuronal homeostasis, mechanisms for supporting mitochondrial integrity and functionality have been explored in patients with HD. Thus, several indicators of oxidative stress (protein carbonyls, malondialdehyde, etc.) were found in the tissues of patients [191]. The pathogenesis of HD is also connected with changes in the antioxidant system [192].

Tabrizi et al. suggested that NO· generation (which is typical for HD) induces a reduction of Aco2 activity, followed by inhibition of complex II/III and activation of the ROS formation cycle with additional inhibition of aconitase, which in turn leads to a significant decrease in the ATP pool. According to the authors, these events are significant for the initiation of neuronal death and can serve as key stages in the development of HD [193].

In general, it has been suggested that the reduction of energy production in mitochondria is a mechanism by which mutant Htt causes the death of neurons [143].

It was shown that although ETC complexes II and III are highly sensitive to inhibition in the conditions of a nitric oxide generation, aconitase appears to be more susceptible to NO negative influence [193]. According to Tabrizi et al. Aco2 oxidation is a reason for enzyme activity reduction in the HD cerebral cortex, putamen, and caudate [193]. Significantly, aconitase dysfunction correlates with superoxide production [23]. As it is known, other mitochondrial enzymes such as citrate synthase or creatine kinase are also oxidized with loss of enzymatic activity in the striatum of patients with HD [194], suggesting the existence of a close oxidative stress connection with the energetic shortage inherent in HD.

When studying the features of mitochondrial functioning in the HD brain, authors [32] found that Aco2 is a substrate of transglutaminase 2 (TGase 2), since rat brain aconitase activity dose-dependently decreased when the organelles were treated with TGase 2. It was found that transglutamination of the enzyme led to the formation of high-molecular aggregates. The authors hypothesized that increased TGase2 activity in patients with HD may facilitate mitochondrial dysfunction by converting Aco2 in the inactive polymers [32] (Figure 6). Thus, the results of Kim et al. indicate that aconitase enzymatic activity can be modulated by oligomeric state in the brain in HD using the enzyme posttranslational modification [32].

Chen and colleagues recognized some regulated proteins in the brain of the Hdh (CAG)150 knock-in mice—an animal model of the early stage of human HD—and aconitase was also among these proteins [195]. The authors demonstrate also the decreased Aco2 protein level and enzyme activity in the striatum of R6/2 mice, the second HD model. Significantly, aconitase activity in the R6/2 mice brain was recovered by N-acetyl-l-cysteine, the famous antioxidant. According to the authors, this study provides Aco2 as a possible marker for the evaluation of the HD status in patients. This data also suggests that Aco2 can be a potential goal in the treatment of HD and indicates that increasing the functional activity of aconitase and/or reducing the intensity of oxidative stress may be an effective component of the treatment strategy for HD. In addition, Aso2 activity can act as an indicator of cell oxidative damage level and may serve as a marker for new antioxidant clinical tests [195].

Significantly, the expression level of ACO2 as a contender marker for the course of HD was confirmed in a study [196]. In particular, Chang et al. showed that this indicator is significantly downregulated in leukocytes of patients that are pre-HD [196].

Authors [197] investigated the relationship between the functional activity of TCA cycle enzymes and HD progress using cultured lymphoblasts from patients with HD. It was shown that measurements of mRNA and protein levels and the activities of the enzymes indicated that aconitase is one of the most significant enzymes in the pathogenesis of HD [197].

It was shown increased iron content in the basal ganglia of patients with HD [198]. In addition, it was demonstrated that the accumulation of iron in the brain of patients that are pre-HD can probably be regarded as a marker of later iron-mediated oxidative stress, leading to changes in the activity of iron-dependent enzymes, especially Aco2 [199].

According to Mena et al. [143], the above data testify in favor of the significant role of mutant Htt in the stimulation of mitochondrial dysfunction, which is likely an early event in the development of HD. Iron accumulation is probably the next event in the pathogenesis of the disease, although this does not exclude the possibility of both processes developing in either parallel or sequential ways (Figure 6).

Thus, it seems that bioenergetic processes in the brains of patients with HD are significantly change, and this is largely mediated by Aco2 activity suppression. Reduced ATP production due to Aco2 dysfunction and huntingtin accumulation may be one of the leading mechanisms in the pathogenesis of HD (Figure 6). Enhancing Aco2 activity or reducing oxidative stress is likely to be an important component of an effective treatment strategy for patients with HD. In addition, aconitase activity indicates the degree of tissue oxidative injury, so the enzyme functional features may be an informative indicator for a promising antioxidant search.

### 3.4. Friedreich’s Ataxia

ND is closely associated with aconitase activity in Friedreich’s ataxia (FRDA). FRDA is one of the most common forms of ataxia, which occurs in 1 in 50,000 people in Caucasian populations [200].

Friedreich’s ataxia is a hereditary ND that is distinguished by such manifestations as progressive impairment of tendon reflexes, limb coordination, dysarthria, nystagmus, diabetes in approximately 20% of cases, and hypertrophic cardiomyopathy. The latter circumstance may likely be an explanation for the fact that a significant proportion of patients with FRDA die from cardiac sequelae [201]. This disease is characterized by progressive destruction of sensory neurons of the peripheral nervous system, especially in the dorsal root ganglia [200].

FRDA is due to mutations on chromosome 9q21, specifically on gene frataxin (FXN) with cytogenetic location 9q21.11. The most general molecular anomaly is a GAA trinucleotide repeat representation in intron 1 of the FXN gene. As is known, healthy human subjects do not have more than 30 trinucleotide repeats, whereas the repeats found in patients with FRDA contain from 70 to more than 1000 triplets [202]. If the mutation present in homozygosis takes place, there is a decrease in the intensity of FXN transcription and a pronounced loss of specific protein functions.

It has been shown that FRDA, like several other NDs, is associated with both mitochondrial abnormalities and oxidative stress intensification [200]. It is a generally accepted fact that the functioning of frataxin is closely related to the formation of ISC. In addition to participating in mitochondrial iron metabolism, frataxin also takes part in reducing the generation of ROS, high concentrations of which are known to lead to increased mitochondrial damage and cell death [202,203] (Figure 6).

The situation is complicated by the typical FRDA decreased aconitase activity, which intensifies the iron accumulation and increases oxidative stress due to the Aco2 dysfunction in the TCA cycle [33,79].

The authors of the study [33] found reduced activity of both aconitase and I-III complexes of ETC in the endomyocardial tissue of FRDA patients. As is known, all four components have ISC, which is damaged due to the formation of ROS when mitochondria are overloaded with iron. According to the authors, deficiency of ICS-dependent enzyme activities in patients was related to mitochondrial iron accumulation and abnormal frataxin, which cause an aconitase and ETC respiratory enzyme dysfunction in FRDA [33].

Now, oxidative stress is considered one of the main promoting factors in the development of FRDA. FXN operates as a partner of Aco2 not only during ISC formation but also under the restoration of oxidatively inactivated aconitase. These views are supported by data on significantly reduced Aco2 activity in patients and animal models of FRDA, which can be the result of a decline in ISC formation and a decline in aconitase activations [39].

Cumulation of iron in the mitochondria of patients with FRDA has been shown mainly in the heart and brain (reviewed in [204]). Increased iron levels correlate with ROS production, which indicates that changes in iron metabolism may promote FRDA development by adjusting the intensity of free radical production. In addition, the latest experimental data from animal models maintain the existence of a mechanism, independent of a free radical generation, that connected iron storage and neuro destruction through the sphingolipid pathway [205].

Huang and collaborators [206], using in experiment a frataxin knockout mouse, showed that frataxin insufficiency consequences in dysregulation of proteins taking part in different iron utilization pathways in mitochondria, such as iron–sulfur cluster synthesis, mitochondrial iron storage, or heme synthesis [206].

Thus, reduction in FXN expression is taken as the principal mover of FRDA development (Figure 6). Frataxin shortages are associated with an iron–sulfur cluster synthesis reduction and the insufficiently effective reparation of oxidatively inactivated Aco2. It is hypothesized that significant Aco2 dysfunction in FRDA patients may result from both the reduction of ISC formation and the reduction of Aco2 activation [38].

### 3.5. Amyotrophic Lateral Sclerosis

Amyotrophic lateral sclerosis (ALS) is a relatively common form of motor neuron disease in adults, which is diagnosed in about 1 in every 50,000 Europeans [207,208].

ALS is an ND that is distinguished by the progressive destruction of large pyramidal neurons of the motor cortex and associated corticospinal tracts, as well as some motor neurons, leading to motor function deprivation, muscle wasting, paralysis, and death [209].

The etiology of most ALS cases stays obscure, but it is generally accepted that the accumulation of free radicals is an important component of the mechanisms of destruction of motor neurons [210].

Mitochondrial abnormalities in ALS are frequently induced by organelle protein mutations [211]. As is known, more than 30 genes have been related to ALS, but mutations in some key genes (SOD1, C9orf72, TARDBP, and FUS) were found in more than half of cases [211,212]. These genes encode proteins involved in most neurons’ functioning, and a combination of these broken protein functions is supposed to facilitate the dysfunction observed in this neuron disease.

SOD1 mutations are one of the well-studied factors in the pathogenesis of ALS [213] (Figure 6). As is known, SOD1 catalyzes the dismutation of •O_2_−, and variations in its functioning could be connected with the development of several NDs as well as ALS [27] (Figure 2). As mentioned earlier (Section 2.3), •O_2_− could disrupt the functions of proteins, including iron–sulfur-containing proteins, particularly Aco2. It is believed that ROS-dependent mitochondrial lesion plays a significant role in ALS pathophysiology [214,215].

Based on the known sensitivity of Aco2 to free radicals’ influence (Section 2.3), it seems that alterations in the SOD1 functioning could radically manipulate aconitase activity. For example, it was shown to have a pronounced decrease in the activity of enzymes isolated from thrombocytes of ALS patients, which the authors associated with impaired functioning of SOD1 [216].

Additionally, Casoni and colleagues [217] showed the sensitivity of Aco2 to ONOO−-dependent tyrosine modification in spinal cord motoneurons of G93A SOD1 transgenic mice—an animal model of disease. According to the authors, this kind of protein modification may be a substantial regulatory event, and ALS can develop when the functioning of the system is significantly impaired.

It was shown [214] that Aco2 activity was essentially reduced in the thrombocytes of ALS patients’ blood samples. Significantly, patients with a higher level of enzyme activity demonstrated greater survival. The authors concluded that Aco2 activity seems to be a substantial factor for the prognosis of ALS patient survival, and the level of Aco2 in the blood can become a promising marker for a prognosis of the course of the disease.

The aconitase activity has also been explored in patient blood samples in other ND. In particular, it is shown that Aco2 activity was decreased in the blood samples of patients with HD and correlated significantly with the disease duration [193,195]. A reduced enzyme activity level has been shown in the blood samples of patients with AD [170].

Currently, the role of iron metabolism in ALS attracts great attention, and several studies have been devoted to investigation of the relationship between changes in iron homeostasis and the pathogenesis of ALS. However, at present, there are certain contradictions and inconsistencies between the research results of various authors. For example, Cheng et al. studies have established that disturbances in bioenergetic processes and iron metabolism may contribute to the development of ALS [218]. At the same time, the authors [219] did not observe a correlative relationship between iron homeostasis and the risk of ALS. Due to insufficient knowledge of the issue, we have marked the possible iron homeostasis contribution to the pathogenesis of ALS with a question mark (Figure 6).

In general, the analysis of the above results indicates that elucidation of the Aco2 participation in the mechanisms of ALS development is the primary task of researchers. Since there is no universal reliable test currently available for ALS, the level of Aco2 activity in the blood can be considered the most promising indicator, the use of which is possible for diagnosing the disease and assessing the therapeutic effectiveness of potential drugs [214].

### 3.6. A Few Examples of Other Types of Neurodegenerative Disorders in Relation to Aconitase Dysfunction

The different variants of the mitochondrial aconitase gene (ACO2) may be responsible for several neurodegenerative disorders, except for those already discussed in this review.

It has been established that mutations in the ACO2 gene on chromosome 22q13 cause a group of neurodegenerative disorders that include optic atrophy-9 (OPA9) and infantile cerebellar-retinal degeneration (ICRD) [138,220]. All registered types of pathologies arose as a result of complex heterozygous or homozygous inheritance of ACO2 variants. However, the pathophysiological features caused by ACO2 dysfunction in patients have not yet been fully characterized [24].

**Figure 7:**
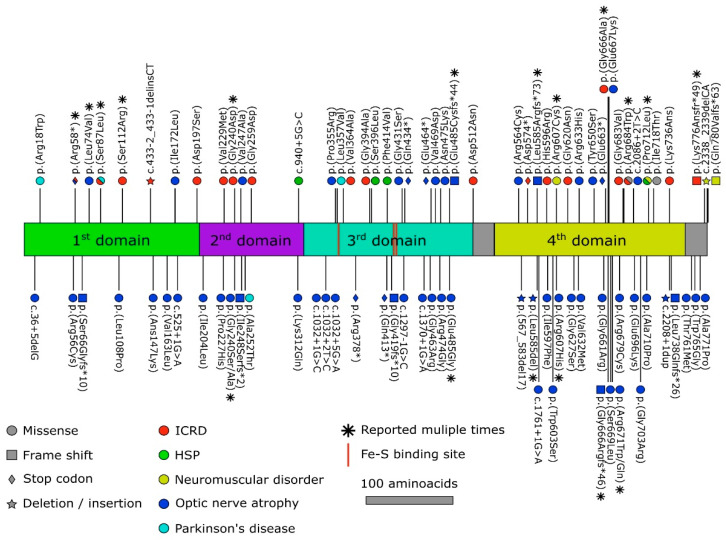
Mutational spectrum associated with Aco2-related disorders in humans. Abbreviation: HSP, hereditary spastic paraplegia; ICRD, infantile cerebellar retinal degeneration. The symbol shapes represent the types of mutations, and the symbol color represents the associated type of a disorder; a black star: a mutation reported more than once.

Spiegel et al. [138] showed that homozygosity mapping followed by whole-exome sequencing detected a Ser112Arg mutation in the aconitase gene (Figure 7), and patients’ lymphoblast Aco2 activity was heavily decreased. According to the authors, a deficiency in Aco2 is associated with an infantile neurodegenerative disorder that mostly touches on the retina and cerebellum. Similar results regarding the c.336C>G (p.Ser112Arg) mutation in the ACO2 are presented in the investigation by Sharkia et al. [221].

Fukada et al. executed whole-exome sequencing of genomic DNA from a patient with progressive cerebellar and cerebral atrophy, seizure disorders, developmental delay, abnormalities in the visual system, and hearing loss [222]. The presence of heterozygous mutations (c.1534G > A, p.Asp512Asn and c.1997G > C, p.Gly666Ala) in ACO2 was detected in patient fibroblasts. According to the authors, reduction of Aco2 activity contributes to dysfunction of the TCA cycle in the patient’s cells, leading to mitochondrial failure, which can lead to symptoms like in mitochondrial diseases [222] (Figure 7).

Neumann et al. [223] established the presence of a heterozygous 51 bp deletion (c.1699_1749del51) in ACO2 in family members with autosomal dominantly inherited isolated optic nerve atrophy. Using patient fibroblasts, the authors found a decrease in aconitase protein levels, while Aco2 enzymatic activity did not differ from the values in control cells of individuals of the same age and sex. Mitochondrial respiratory activity was reduced in patient-derived cells. In addition, a decrease in the copy number and transcription level of mtDNA was found in mutant fibroblasts. The authors conclude that mutation in ACO2 is enough to facilitate mitochondrial abnormalities and increased sensitivity to oxidative stress as leading causes of cell death associated with optic nerve atrophy [223].

According to Charif et al. [224], biallelic mutations in ACO2 were found in patients with neurodegenerative syndromes, including recessive optic neuropathies (locus OPA9) and infantile cerebellar retinal degeneration. In European groups of individuals with genetically undecided optic neuropathies, authors recognized more than 60 cases harboring ACO2 variants, 50 of which carried dominant mutations, highlighting the important contribution of pathogenic ACO2 variants to dominant optic atrophy. A study of skin fibroblasts from patients with ACO2 mutations allowed to establish a decrease in aconitase enzymatic activity as well as impaired mitochondrial respiration. It was shown by the authors that ACO2 is one of the most often mutated genes in autosomal inherited optic neuropathies and also emphasized the key involvement of the aconitase-dependent stage of the TCA cycle in ensuring the viability of retinal ganglion cells [224].

As noted earlier, the pathogenic variants of ACO2 are responsible for a range of pathologies, including different forms of optic nerve degeneration, from isolated optic neuropathy to complex neurodegenerative syndromes. Authors [225] noted that some syndromic patients do not have optic neuropathy, which they believe supports the classification of c.220C>G and c.336C>G variants as likely pathogenic. In general, these data allowed the authors to suggest that the clinical manifestations of ACO2 variants should be considered as a continuum of symptoms, and the classification of some relatively common pathological condition requires clarification [225].

Some mutations have been reported to change the Aco2 enzymatic functioning in a PD. Zhu et al. [28] collected the GWAS sequencing data from several genetic studies using whole-genome, whole-exome, and targeted panel gene sequencing technologies, which involved a group of patients with PD and a control group with about 1500 in each. The authors found that four variants, Arg18Trp, Ser87Leu, Ala252Thr, and Leu357Val, cause Aco2 activity decline (Figure 7).

Thus, it seems that inactivation and dysfunction of Aco2 due to pathogenic variants of the ACO2 gene could promote the development of various types of ND. One can probably conclude that with an insufficient number of effective noninvasive markers of ND, determination of the ACO2 gene sequence or Aco2 activity in lymphoblasts (or another type of cell) may be justified and effective in similarly affected individuals, based on clinical evidence [138].

## 4. Conclusions

Studies presented in this review have shown that mitochondrial aconitase is an intriguing enzyme that may contribute to the progress of oxidative stress-dependent body conditions such as aging and ND.

It has been suggested that the existence in the eukaryotic mitochondria of an oxygen-sensitive aconitase isoform Aco2 provides a feedback mechanism regulating the production of free radicals during mitochondrial respiration by slowing the TCA cycle and decreasing electron flux through the ETC and, as a result, the production of the mitochondrial-derived •O_2_−. However, under some pathophysiological conditions, such as neurodegenerative disorder, a rise in the rate of ROS generation may cause an irreversible inactivation of aconitase. As a result, the inactivation of the enzyme may significantly slow down normal electron flow to oxygen. It can cause an enlarged generation of ROS, thus initiating the vicious cascade of ROS production and subsequent increase in oxidative damage to a broad range of macromolecules. Dysfunctional mitochondrial aconitase is now considered an important part of the pathogenesis of several neuronal disorders.

In general, the literature analysis presented in this review indicates that a great body of evidence has been displayed that dysfunctional Aco2 is one of the key factors that could stimulate PD, AD, HD, FRDA, and ALS.

In addition, mutations in the ACO2 gene on chromosome 22q13 were recognized in patients afflicted with a lot of pathological conditions, from progressive cerebellar/cerebral atrophy and optic nerve atrophy to intellectual disabilities and hearing loss.

Thus, the literature data presented in this review generally indicate that mitochondrial aconitase is engaged in the regulation of cellular metabolism and iron homeostasis, equilibrates the regulatory and damaging effects of ROS, and can be one of the important contributing factors to oxidative stress-dependent conditions, like neurodegenerative diseases. Although Aco2’s specific role in neurodegenerative conditions development remains not fully understood, the fact is already obvious that increasing the efficiency of cell energy production by modulating Aco2 activity can be considered a promising therapeutic approach that can at least delay the progressive damage of neurons during the development of neurodegenerative diseases.

## Data Availability

No new data were created or analyzed in this study. Data sharing is not applicable to this article.

## Abbreviations

Aco2	mitochondrial aconitase
ACO2	mitochondrial aconitase gene
AD	Alzheimer’s disease
ALS	amyotrophic lateral sclerosis
APP	amyloid precursor protein
ETC	electron transfer chain
EPR	electron paramagnetic resonance
FRDA	Friedreich’s ataxia
FXN	frataxin
GWAS	genome-wide association studies
HD	Huntington’s disease
IRP1	iron response proteins 1
IRE	iron regulating element
ISC	iron–sulfur clusters
mtDNA	mitochondrial DNA
MtMP	mitochondrial membrane potential
ND	neurodegenerative disease
PD	Parkinson’s disease
OXPHOS	oxidative phosphorylation
ROS	reactive oxygen species
RNS	reactive nitrogen species
SN	substantia nigra
TCA	tricarboxylic acids cycle
UTR	untranslated region of mRNAs
